# Analysis of Sampling Bias in Large Health Care Claims Databases

**DOI:** 10.1001/jamanetworkopen.2022.49804

**Published:** 2023-01-06

**Authors:** Alex Dahlen, Vivek Charu

**Affiliations:** 1 Quantitative Sciences Unit, Department of Medicine, Stanford University School of Medicine, Stanford, California; 2 Department of Pathology, Stanford University School of Medicine, Stanford, California

## Abstract

This cross-sectional study characterizes variation in sampling in a large health care claims database at the zip code level in 2018 and assesses whether socioeconomic and demographic factors are associated with inclusion.

## Introduction

Health care claims databases that aggregate claims from multiple commercial insurers are increasingly being used to generate clinical evidence.^1,2,3^ These databases represent a nonrandom sample of the underlying population, but often little attention is paid to the inherent sampling bias within the data, and how it might affect results. As an illustrative example, we characterize variation in sampling in the Optum Clinformatics Data Mart (CDM) at the zip code–level in 2018 and identify socioeconomic and demographic factors associated with inclusion.

## Methods

This cross-sectional study was approved by the Stanford University institutional review board. Reporting followed the STROBE reporting guideline.

The Optum CDM consists of administrative claims derived from several large commercial and Medicare Advantage health plans. For the primary analysis of this cross-sectional study, we count the number of individuals with CDM coverage on a single day (June 1, 2018) in each zip code and compare it with the 2018 census estimates of the total population in that zip code. In sensitivity analyses, we also consider 1 strictly larger cohort—individuals with at least 1 day of coverage at any point during 2018—and 1 strictly smaller cohort—individuals with continuous coverage during the entire year of 2018.

To explain the variation in zip code–level CDM sampling, we fit an inverse-variance weighted multivariable linear regression model with 30 socioeconomic and demographic features extracted from the 2018 census, and state-level fixed effects. See the Table for the list of features and the eAppendix in Supplement 1 for details about the model and methods (including sensitivity analyses).

**Table.** Associations Between 30 Socioeconomic and Demographic Features and Claims Database Sampling Fraction at the Zip Code Level, Accounting for State-Level Variation in Sampling Fraction in 2 Models

Characteristic	%		*P* value
	Partial correlation coefficient^a^	Multivariable regression coefficient (SD)^b^	
Population			
Total population (millions)	0.01	−0.0005 (0.00018)	<.001
Log_10_ pop density (1/square mile)	0.14	0.67 (0.06)	<.001
Female sex	0.12	0.050 (0.010)	<.001
Race and ethnicity			
Asian (non-Hispanic)	0.16	−0.010 (0.004)	<.001
Black (non-Hispanic)	−0.15	−0.008 (0.002)	<.001
Hispanic	−0.19	0.001 (0.004)	.64
White (non-Hispanic)	0.20	[Reference]	NA
Other^c^	−0.08	−0.026 (0.008)	<.001
Age, y			
<18	−0.09	[Reference]	NA
18-40	−0.21	−0.019 (0.010)	<.001
40-60	0.32	0.084 (0.014)	<.001
60-80	0.14	0.002 (0.012)	.71
>80	0.12	0.055 (0.024)	<.001
Household income, $			
<15 000	−0.34	[Reference]	NA
15 000-30 000	−0.40	−0.016 (0.014)	.01
30 000-45 000	−0.37	−0.009 (0.012)	.16
45 000-60 000	−0.23	0.002 (0.014)	.78
60 000-100 000	0.10	0.007 (0.010)	.15
100 000-125 000	0.33	0.033 (0.018)	<.001
125 000-200 000	0.43	0.016 (0.012)	.01
>200 000	0.42	0.071 (0.014)	<.001
Work and insurance			
Unemployed	−0.29	−0.001 (0.014)	.89
No health insurance	0.31	−0.030 (0.008)	<.001
Education			
Less than high school	−0.33	[Reference]	
High school	−0.27	0.038 (0.01)	<.001
Some college	−0.09	−0.010 (0.008)	.01
College	0.41	0.071 (0.010)	<.001
Graduate	0.30	−0.021 (0.010)	<.001
Housing			
Houses that are owner occupied	0.25	−0.003 (0.004)	.14
Median house price (millions), $	0.36	−0.00001 (0.00003)	.43

Abbreviation: NA, not applicable.

^a^ The first set of models considers each covariate of interest separately, along with state-level fixed effects. Partial correlation coefficients derived from this model are presented; positive correlations indicate that zip codes with higher values of the covariate of interest are associated with higher zip-code level sampling in the claims database, even after adjusting for state-level clustering in sampling.

^b^ The second model is a full multivariable model that includes all 29 covariates of interest in addition to state-level fixed effects. For example, for a 10 percentage increase in a zip code’s fraction of households earning greater than $200 000, the model suggests the claims database sampling fraction will increase by 0.6 percentage points, on average.

^c^ Other race and ethnicity includes persons identifying as non-Hispanic American Indian and/or Alaska Native, non-Hispanic Native Hawaiian and Other Pacific Islander, non-Hispanic other races, and 2 or more races.

## Results

There were 16.4 million distinct individuals captured in the CDM on June 1, 2018, representing 5.4% of the US population. The median (IQR) zip code sampling fraction was 4.4% (2.5%-7.1%), with clear geographic variation in sampling (Figure). At the state level, Alaska had the lowest sampling rate (0.8%) and Colorado had the highest sampling rate (11.0%); in multivariable regression models, state-level fixed effects explained 34.6% of the zip code–level variation in sampling.

**Figure.**
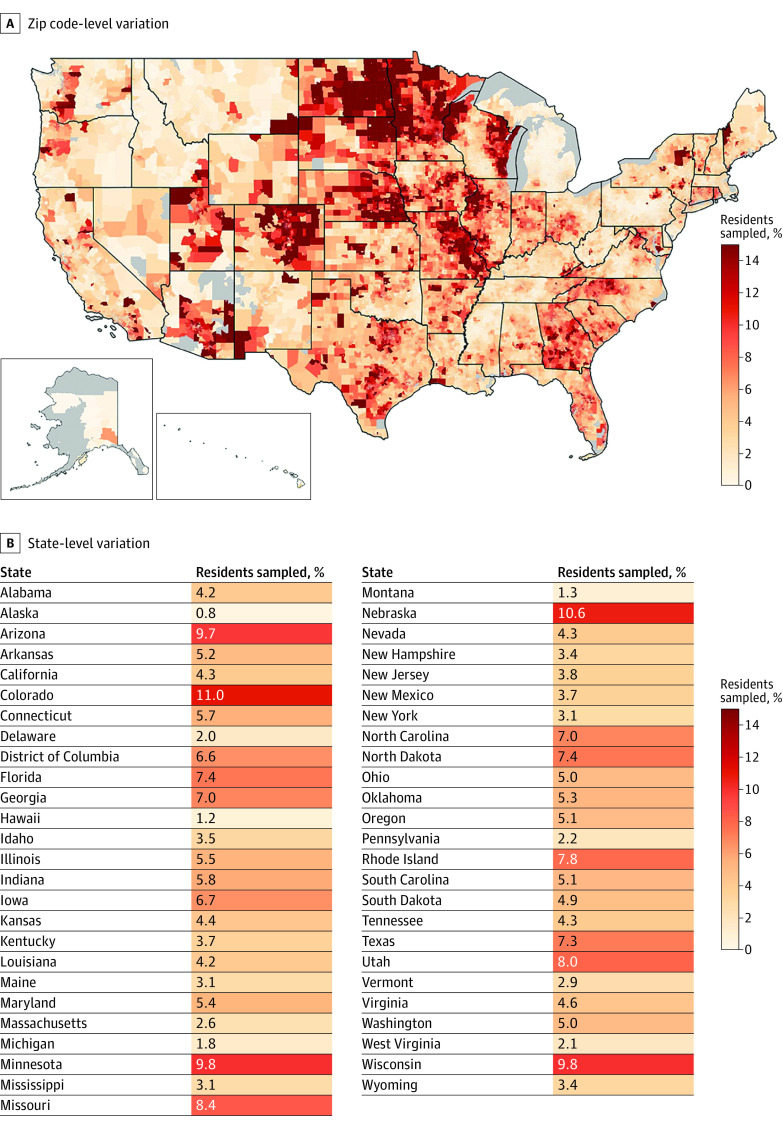
Zip Code–Level and State-Level Variation in Sampling in the Optum Clinformatics Data Mart Database (CDM) in 2018 A, Clinformatics Data Mart sampling in each zip code, estimated from the number of patients with coverage in CDM on June 1, 2018. B, Clinformatics Data Mart sampling in each state, estimated using the same criteria.

Associations between socioeconomic and demographic features and CDM sampling fraction, after adjusting for state-level variation, are provided in the Table. Estimated partial correlations and regression model coefficients found that inclusion in CDM was associated with zip codes that had wealthier, older, more educated, and disproportionately White residents. These patterns were robust to the choice of cohort definition, and across the 10 most populous states individually. Socioeconomic and demographic features explain an additional 19.4% of the zip code–level variation in sampling on top of state-level variation, for a total adjusted *R*^2^ of 54.0%.

## Discussion

To interpret results generated from health care claims databases, it is essential to understand which patients are represented in them. We found that inclusion in the Optum CDM at the zip code level in 2018 varies spatially and along socioeconomic and demographic lines. Our study is limited by the granularity of demographic data; we analyzed data at the smallest geographic scale available in CDM, the zip code. Given our findings, there is likely to be additional bias within zip codes as well.

The socioeconomic and demographic features correlated with overrepresentation in claims data have also been shown to be effect modifiers across a diverse spectrum of health outcomes.^4^ The combination of heterogenous sampling and effect modification—both driven, in this case, by social determinants of health—gives rise to external validity bias, where results generated from the claims data will fail to generalize to the underlying population.^5^ This bias can affect studies that estimate disease incidence and/or prevalence and even comparative effectiveness studies that use contemporary causal inference methods whenever there is meaningful heterogeneity in treatment or policy effects along socioeconomic and demographic lines.

Our study highlights the well-established importance of investigating sampling heterogeneity in analyses of large health care claims data to evaluate how sampling bias might compromise the accuracy and generalizability of results.^6^ Importantly, investigating these biases or accurately reweighting the data will require data external data sources outside of the claims database itself. Health care claims databases offer enormous promise for medical research; characterizing and overcoming sampling bias in these data sets is essential.

## Notes

Supplement 1. **eAppendix.** Supplemental Methods

Supplement 2.
Data Sharing Statement
