# Supplementary material for: Analysis of Sampling Bias in Large Health Care Claims Databases
Source: JAMA Netw Open. 2023 Jan 6;6(1):e2249804. doi: 10.1001/jamanetworkopen.2022.49804 (PMC9857613; doi:10.1001/jamanetworkopen.2022.49804)
Supplement: Supplement 1. — eAppendix. Supplemental Methods [file jamanetwopen-e2249804-s001.pdf]

Supplemental Online Content

Dahlen A, Charu V. Analysis of Sampling Bias in Large Health Care Claims Databases. *JAMA Netw Open.* 2023;6(1):e2249804. doi:10.1001/jamanetworkopen.2022.49804

eAppendix. Supplemental Methods

This supplemental material has been provided by the authors to give readers additional information about their work.

© 2023 Dahlen A et al. *JAMA Network Open.*

eAppendix. Supplemental Methods

Here we describe additional details about the methodology used.

Optum's Clinformatics® Data Mart Database (CDM) is a de-identified database derived from a large adjudicated claims data warehouse. We considered three cohort definitions: (1) all unique patients in CDM on June 1, 2018; (2) all unique patients with at least a single day of coverage from January 1, 2018 to December 31, 2018 and (3) all unique patients with coverage during the entire year, January 1, 2018 to December 31, 2018. Cohort 1 parallels a common use of the that computes incidence or prevalence rates by normalizing counts by patient-days of coverage; cohort 2 is the maximal count of covered patients in 2018; and cohort 3 parallels a different common use of the data where cohorts with longer periods of continuous coverage are compared.

For each cohort definition, we estimated the zip-code level CDM sampling fraction as the number of unique patients in each zip code in CDM divided by the number of unique persons in the American Community Survey (ACS) 2018 5-year census. Of note, CDM aggregates some smaller zip-codes into groups; all analyses are performed at the level of CDM's zip-code clusters definition.

Socioeconomic/demographic information was derived from the ACS 2018 5-year census, and included population density; the fraction of persons self-identifying as female, Asian, Black, Hispanic, White, or other races; the fraction of persons aged <18, 18-39, 40-59, 60-19, and >80; the fraction of households earning (in US dollars) less than $15,000, $15,000-$30,000, $30,000-$45,000, $45,000-$60,000, $60,000-$100,000, $100,000-$125,000, $125,000-$200,000, and over $200,000; the fraction of individuals unemployed and the fraction of individuals without health insurance; the fraction of individuals with who completed less than high school, high school, some college, college, and graduate school; the fraction of houses owner-occupied; and the median house price. This data was read in at the census track level and then to get the data on equal footing, we rolled the census data up using a population-weighting or square mileage-weighting, as appropriate.

The multivariable model is a linear regression of the form:

$$E[Y_i \mid \mathbf{X_i}, \mathbf{Z_i}] = \beta_0 + \boldsymbol{\beta}_{1\text{-}25} \cdot \mathbf{X_i} + \boldsymbol{\beta}_{26\text{-}76} \cdot \mathbf{Z_i},$$

where *i* indexes each zip-code, Y_i is the CDM sampling fraction in each zip code, $\mathbf{X_i}$ represents a vector of 25 census features for each zip code (30 total socioeconomic and demographic features with 4 comparisons groups) and $\mathbf{Z_i}$ represents a vector of 50 state-level fixed effects (51 total states including the District of Columbia with one comparison group). Each zip code is weighted by the inverse of the variance associated with the zip code level estimate of CDM sampling. We used the standard binomial estimate of variance, modified by Winsorizing the estimate of the sampling fraction at the 5th percentile, to prevent unstable weighting. Thus, the weights used scale with the total census population in each zip-code, down-weighting the contribution of very

© 2023 Dahlen A et al. *JAMA Network Open.*

small and high variance zip codes. We produced standard diagnostic plots for this model, which demonstrated a reasonable fit.

Partial correlation coefficients were computed using the analogous short model with only a single census variable, in addition to the 50 fixed effects. The analysis was carried out in Python version 3.8.5, and the code to reproduce this analysis is publicly available at: https://github.com/alex-dahlen/who_is_in_optum.

© 2023 Dahlen A et al. *JAMA Network Open.*
