# Supplementary material for: Analysis of Sampling Bias in Large Health Care Claims Databases
Source: JAMA Netw Open. 2023 Jan 6;6(1):e2249804. doi: 10.1001/jamanetworkopen.2022.49804 (PMC9857613; doi:10.1001/jamanetworkopen.2022.49804)
Supplement: Supplement 2. — Data Sharing Statement [file jamanetwopen-e2249804-s002.pdf]

Data Sharing Statement

Dahlen. Analysis of Sampling Bias in Large Health Care Claims Databases. *JAMA Netw Open*. Published January 06, 2023. doi:10.1001/jamanetworkopen.2022.49804

Data
Data available: No

Additional Information
Explanation for why data not available: Optum CDM data is not publicly available, unfortunately.
